# Erosion of the Teeth

**Published:** 1893-04

**Authors:** Safford G. Perry

**Affiliations:** New York


### EROSION OF THE TEETH.[1]

BY SAFFORD G. PERRY, D.D.S., NEW YORK.

IN the development of dental theory and practice, it is fair to assume that those questions which are easiest are the ones that are settled first. It follows, of course, that those which are hardest must be settled last.

It took a long time, and engaged the earnest efforts of acute observers in all parts of the world, before the cause of dental caries was understood. It took even a longer time before it was possible to account for the severe inflammations that often occur about the roots of dead teeth.

The diversity of views in reference to the cause of erosion of the teeth is conclusive evidence that it is not yet finally decided. The conditions are such that it cannot be an easy question to settle.

It is not surprising that there should have been, and should still

---

[1] Read before the New York Odontological Society, January 17, 1893.

be, such a diversity of opinion as to its etiology. Probably no question in dental practice has been written upon or discussed with such a feeling of uncertainty as to the truth of the particular theory advanced. It must have been perceived that those who have written or spoken on the subject during the last ten or fifteen years have felt that they were groping in the dark; and if they seemed to believe they were not, there was yet such a lack of converging and convincing evidence in support of their particular theory that it is safe to say by common consent the question of the cause of erosion is considered still unsettled.

The earliest observers, exercising their common sense, believed it due to acid action. The latest authorities, profiting by an acquaintance with all the observations that have accumulated, have gone no further.

All along the line, while there has been a preponderance of belief in acid action, there has been, so far as I have been able to learn, no demonstration and no agreement as to what the acid is, or as to what are the conditions, constitutional or otherwise, that produce it.

Without taking the time, in an impromptu discussion of this kind, to give the different theories that have been advanced from time to time, I believe this will be accepted as a fair statement.

In the very nature of the case, until we are able to apply a more accurate system of tests and employ a more trustworthy and convincing method of observation, we shall have to accept, as has been done in the past, such a theory as seems in accord with the largest number of facts we are able to obtain. Each person who has spoken or written on the subject, up to the present time, has very naturally adopted and advanced that theory which seemed to him to account most satisfactorily for the conditions he had observed.

I will now give my idea of the cause of erosion, but you will doubtless perceive something of the same feeling of uncertainty that I have alluded to as characterizing the opinions of others. I am not able to produce facts enough to entitle me to speak with scientific assurance, but I have accumulated evidence which seems to me so reasonable and so consistent that I shall venture to formulate a "theory."

I know that science deals with facts, not theories, and speculation is somewhat out of place in scientific discussion, but in the absence of positive knowledge I think its employment is a legitimate means of searching after truth. It has always been employed more or less by scientists, and naturally so, for all down the ages, men, when brought face to face with the unknown, though they have felt like helpless children, have yet craved an explanation of what they could not understand, and, in their inability to attain this, have derived some satisfaction in accepting as true that which seemed to be true. Many of the important truths of medicine have had to be accepted first in this way. If, in the language of the poet, "it is better to have loved and lost than never to have loved at all," so I think it may be better to accept a theory which yet may be destroyed than to have no theory at all.

In our daily practice it may serve a good purpose, as it may give us the courage to adopt a positive line of treatment, and it may infuse an indefinable confidence that in itself may help us to attain results that might not be reached if, not knowing our own minds, we drift about like a ship at sea without a rudder. There is still another reason why it is better to accept and work upon a theory, and that is that it may prove to be a true one.

Whether the theory of erosion which I shall advance to-night will stand the glare of the search-light remains, of course, to be seen. It is one which is not entirely original with me, as it has already been advanced, as I shall show; but yet it is one which I arrived at in an original and independent manner through the accident of personal experience, and so many years ago that I now feel entitled to speak with some confidence about the observations I have made.

I believe erosion of the teeth is due to an excess of uric acid in the blood, and the reasons for coming to this conclusion will be best given by the relation of the following personal experience:

Some years ago my attention was drawn to a peculiar condition on the labial surfaces of my incisors, not noticeable when I looked in the glass, but easily felt with the tongue. It was the minute beginning of that condition we know so well as erosion.

At the same time I was greatly annoyed by an eczematous condition of the skin, which manifested itself upon the hands and feet. The first appearance of this condition was marked by little aggregations of blebs, each bleb not larger than the head of a pin, which prickled and itched, and which, when punctured, exuded a watery fluid.

These blebs extended over little surfaces half as large as a ten-cent-piece, and, coalescing as they burst, ran into each other, making eczematous patches, which, on healing and disappearing after two or three weeks, would be followed by others of the same kind, which ran the same course on some other part of the hands, fingers, or feet. They became very marked and persistent, particularly between the toes of the right foot, on which, in operating, I stood most.

The explanation given for this was that the condition was partly a neurotic one, and might be expected to manifest itself on the extremities most deficient in nerve-force. The condition of the hands became a source of alarm, as it can be readily seen that it was not agreeable to operate with three or four patches of court-plaster in constant use to hide the condition of the skin.

I put myself under treatment at the hands of a well-known specialist, and at the same time looked up the literature on eczema most carefully. The treatment proved to be beneficial, but did not produce a prompt cure.

Later on, upon my own responsibility, in the hope of better satisfying myself in my own way if this condition was dependent on an acid or an alkaline food-supply, I first tried for weeks the faithful use of alkaline mineral waters, and then for weeks the equally faithful use of acids,—as vinegar and lemon-juice,—but with no very marked result.

Remembering as a boy that good sportsmen kept their dogs from having the mange by withholding meat from them, and believing that this condition of the skin was something like that disease, I abandoned meat entirely, and lived mostly on milk. After a few weeks this eczematous condition gradually disappeared, and to this day has never to any great extent returned.

That this cure was accomplished solely by the abandonment of meat I do not now believe, for I was still under treatment to a certain extent, and I was convinced, as my physician claimed, that the condition was partly neurotic, and that less work, more rest, and more exercise in the open air, accompanied by times of deep breathing to insure a fuller oxygenation of all food-elements, were important factors in producing a cure.

At about the time this eczematous condition was at its worst, I was awakened one morning before daylight with a dull pain in the region of the kidneys. This increased steadily until it became indescribable,—language is weak when used to express its intensity. Morphine was used in large doses, but not promptly enough to save me from the memory of a frightful experience.

At about eleven o'clock relief came instantly, caused by the dropping from one of the ureters into the bladder of a renal calculus, which, when recovered from the urine, was found, of course, to be a nodule of uric-acid crystals.

Here was a new disclosure, and further medical advice elicited the fact that I must consider myself as belonging to those who have the lithæmic diathesis, and that uric acid must be considered my greatest enemy.

A still more careful study on my part of the real cause of the eczematous condition brought out more clearly than I had before known the fact that uric acid was also the most potent factor in its production. Dr. Bulkley tells me there can be no doubt of this.

I was therefore on the verge of gout, other evidence of which was shown by the stiffness of the joints and the pain that was almost constant in them.

Once in possession of this idea of the real cause of my troubles, the means of cure became more clearly comprehended. Since I live in-doors and lead a sedentary life, it consisted in lessening the amount of food taken, and in greater care in its selection; being careful to reject the acid-forming elements, such as sweet wines, champagne, beer, food too rich in starch, and sweets in general,—in fact, all rich foods that overburden the liver and prevent it from fulfilling its function of liberating the waste products which shall be ultimately thrown off in the form of soluble urea.

That excessive meat-eating is productive of impeded liver-action and the formation of an excess of uric acid there can be no doubt.

If one lives in the open air and takes active and constant exercise, the selection of food may not be of so much consequence, and meat can be taken to advantage and without harm; but not so if the life is a sedentary one, and is passed in the warm atmosphere of a close room. In proof of this I can do no better than to quote the following rather startling sentences from a remarkable book called "The Town-Dweller," written by that brilliant and lamented English physician, Dr. J. Milner Fothergill. He says, "The town-dweller is more or less dyspeptic. The food which the countryman can digest is beyond his powers. Meat—the flesh of animals —sits easiest on his stomach, and gives him the least discomfort; therefore he prefers it to other food, and, what is more, can easily procure it. It is tasty, too, and gratifies his palate. Why should he not indulge in it?

"Unfortunately, there is something more about meat than its ready digestibility by a feeble stomach. Its resultant products have to be got rid of, and be cast out of the body. In birds and in reptiles the form of excretion is uric acid. When the mammalia are reached, we find a fluid urine, and the form of excretion the soluble urea. But a little of the early uric-acid formation clings to all of us. Indeed, the embryo has the uric-acid formation as a matter of fact. . . . The conversion of waste, effete, and nitrogenized bodies into uric acid and urea is one of the functions of the liver. The soluble urea is the more highly oxygenized substance of the two. When a person with a competent liver lives too well, year after year, he gets the gout,—rich man's gout. But when a person comes into the world with a feeble or insufficient liver, the viscus is liable to revert or fall back upon the embryonic formation,—viz., uric acid.

"In such cases it is very common to find gouty phenomena, even with a very spare dietary; and this is known as 'poor-man's gout.' For long this form of gout, or rather lithiasis, was the opprobrium of the medical profession; but recent research has revealed its real nature. It is now known to be due to deficiency of action of the liver. The liver is one of the deprivates of the hypoblast; and the town-dweller, in addition to a feeble stomach, has an incompetent liver. The meat which he eats to ease the labors of his stomach overburdens his liver. He avoids the stomach-ache, but in doing so drives his liver downward to the uric-acid formation.

"Lithiasis is his lot, and the urine contains quantities of lithates. This is a serious matter, as the comparatively insoluble uric acid is injurious to kidneys constructed to eliminate the soluble urea. A slow, chronic, interstitial inflammation goes on in the kidney, which spreads bit by bit throughout their structure, and ultimately works their ruin. The condition is best known as "chronic Bright's disease," and this form of kidney-change is variously known as the 'cirrhotic,' 'granular,' 'gouty,' or 'contracted' kidney.

"The kidney-change is, however, but one outcome of the morbid process set on foot by lithiasis. The circulating organs are modified in the great vaso-renal change started by constant presence of uric acid in the blood. The heart enlarges, while the arteries harden, and death commonly enough takes place from mischief in the circulation before the kidneys attract attention. The vaso-renal change has usually been on foot some considerable time before the kidney is seriously damaged. But sooner or later—if the patient lives—renal mischief becomes obvious.

"The meat-eating town-dweller is specially liable to chronic Bright's disease in consequence of his overtaxing a naturally insufficient liver. But there is still another factor in the case.

"It has been said before that uric acid is less highly oxygenized than urea. If the embarrassed liver could have the help of unlimited oxygen, it could struggle more successfully with the waste and excrementitious matters, and burn them up into the soluble urea.

"But, as has been pointed out, the air breathed by the town-dweller is defective in oxygen, and absolutely lacking in ozone. The town-dweller burns the candle at both ends.

"He gives his liver too much to do, and then handicaps it by compelling it to do its work with a defective supply of oxygen. No wonder, then, that his liver reverts to the uric-acid formation of the Saurian in his tropical swamp. The wonder would rather be, if the liver could do its work properly under such unfavorable circumstances. Lithiasis, then, with all its consequential and resultant train of maladies, is the lot of the town-dweller,—of the man who has divorced himself from the soil and the ozonized air which blows over it. Liver-insufficiency with unsuspected kidney-injury is often the reason why individuals go down under the onslaught of disease, as we shall see shortly."

Another great authority, Sir Henry Thompson, in a valuable little book entitled "Diet in Relation to Age and Activity," in discussing the question of overfeeding and improper selection of food, says, "I have for some years past been compelled by facts which are constantly coming before me, to accept the conclusion that more mischief in the form of actual disease, of impaired vigor, and of shortened life, accrues to civilized man, so far as I have observed in our own country and throughout Western and Central Europe, from erroneous habits in eating than from the habitual use of alcoholic drink, considerable as I know the evil of that to be."

Further on, in considering food in its relation to the gouty and rheumatic tendency, which of course is the uric-acid tendency, he says, "The accumulated store of aliment—the unspent food, so to speak—which saturates the system is happily often got rid of by those special exercises to which so large a portion of time and energy is devoted by some people. It is to this end that men at home use dumb-bells or heavy clubs, or abroad shoot, hunt, and row, or perform athletic and pedestrian feats, or sweat in Turkish baths, or undergo a drench at some foreign watering-place,—all useful exercises in their way, but pursued to an extent unnecessary for any other purpose than to eliminate superfluous nutrient materials, which are occasioning derangement in the system, for which these modes of elimination are sometimes an efficient cure, and thus are often ordered by the medical adviser.

"But, as we increase in age—when we have spent, say, our first half-century—less energy and activity remain, and less expenditure can be made; less power to eliminate is possible at fifty than at thirty, still less at sixty and upward. Less nutriment, therefore, must be taken in proportion as age advances, or rather as activity diminishes, or the individual will suffer.

"If he continues to consume the same abundant breakfasts, substantial lunches, and heavy dinners which at the summit of his power he could dispose of almost with impunity, he will in time certainly either accumulate fat or become acquainted with gout or rheumatism, or show signs of unhealthy deposit of some kind in some part of the body, processes which must inevitably empoison, undermine, or shorten his remaining term of life."

Associating the signs of erosion in my incisors with these conditions of excess of uric acid, and noting that as the uric-acid manifestations by proper care and treatment lessened or disappeared the erosion stopped, I felt that I had reason to believe that here at last was a more reasonable explanation of this condition than we had yet had.

At the very first it seemed a reasonable one, for the gouty condition and the period of erosion rarely occur till advanced adult life, and then I remembered that I had often observed that erosion was most common among those patients who were most indolent and best fed. In fact, I had sometimes thought that it was most common among ladies in high society, whose lives were mostly passed in-doors and subject to the temptations of luxurious living.

I have never had the opportunity for the observation of the teeth of many of the working-classes whose lives have been spent mostly in the open air, and who are least likely to be overfed, but in such cases as I have seen I never remember to have noticed a case of pronounced erosion. But, on the other hand, I think with this class accumulations of tartar are rather common. In cases of erosion I feel certain that extensive accumulation of tartar does not occur. It is deposited to some extent, but the acid reaction of the mouth does not favor its accumulation.

For many years, before I had suspected what I now consider the cause of erosion, I thought it to be a condition most common to the luxurious classes. In fact, I think by common consent it has come to be regarded as peculiarly a disease of modern high civilization.

Commencing to make observations among my patients, I came quite frequently upon cases that seemed to confirm the idea I have expressed. All cases of erosion, however, did not give evidence of the existence of established gout; but since I have commenced to make these observations, I never yet remember to have seen a case where it was not found that there was little care in the selection of food, and where there was not evidence that the life was passed without much thought of those conditions that are necessary to good health.

Besides, it does not follow, by any means, that the presence of an excess of uric acid indicated a condition of gout, although it may be laying the foundations on which the superstructure of that disease will yet be raised; or it may manifest itself in disturbed and deranged conditions which are not recognized or considered as gout at all.

I think in every instance of marked and advanced cases of erosion there was evidence of the existence of gout.

On the other hand, it must be said that one will see positive cases of gout where there are no signs of erosion. Many cases of erosion gave evidence of conditions which had not been suspected to be of a gouty nature, and yet these were unmistakable.

The worst case of erosion I ever saw was in the mouth of a gentleman who nearly every summer is sent to Carlsbad, in the language of his physician, "To keep down the alarming excess of uric acid in his blood."

Another, whose teeth were attacked by most extensive erosion on nearly all their surfaces, was a martyr to gout, and finally died suddenly from complications arising from that disease.

Becoming more convinced of the truth of this idea, I had for several years many conversations with Dr. Darby on the subject, and I was still more convinced by finding that his thoughts had for some time also been tending in this direction. He was so impressed with the truth of this idea that he prepared a paper on the subject, which he read at Albany last May. The paper was illustrated by a series of casts, which gave, in my estimation, unmistakable evidence of the soundness of this theory.

Some of the discussions that were then brought out gave strong support to the theory. It was also made clear that others had for some time inclined to this view; and I learned there that Dr. Kirk, the year before, had expressed the belief that "erosion, strictly so called, of the teeth is, in all probability, a pathognomonic symptom of the gouty or rheumatic diathesis." In the discussion of Dr. Darby's paper, Dr. L. A. Faught said, "When Dr. Perry made the assertion that we should examine for uric acid, he touched upon the line of subject that I was at work upon. We should look to the cause, and when we find this uric acid condition the analysis of the urine would often throw light upon it. I have been engaged in that study—not directly from the stand-point of erosion, but in relation to the production of uric acid—for some time. There is one thing I have been waiting to hear expressed, and that is that you will find in the majority of cases where you have the condition of erosion what is known as a bilious temperament."

Dr. K. C. Gibson, in continuing the discussion, said, "I am a victim of gout, and have been for twenty-five years. I have found that not only through an examination of the urine, but also of the blood. The erosion which has taken place on my teeth is a guide to me in treatment.

"Whenever the eroded teeth become sensitive to the nail of my finger, I place myself under treatment.

"My physicians tell me that an examination of the blood has been of more assistance to them in my case than the examination of the urine."

Other gentlemen also gave testimony of a similar nature.

In support of my theory I could give a list of cases that would exhaust your patience. I will only refer to a few of them.

A well-known New York lawyer came to me after about a year's absence, and I said to him, "You have an excess of uric acid. I am sure of this from the marked erosion of the teeth that has occurred since I saw you." His reply was, "Yes, my physician is treating me now for an excess of uric acid, and is alarmed at my condition."

One of the most intelligent women I ever knew came to me several years ago, in despair over the rapid erosion that was going on across the front surfaces of the incisors. I preached this doctrine of uric acid to her, and indicated as well as I could what reform must be made in diet and in the mode of living. Acting on this advice, in a short time the action ceased, and the eroding surfaces lost their polish and their sensitiveness, and her general health improved.

Yesterday she was in my office, and said she was an enthusiastic convert. She further said that her teeth were like a thermometer, and were quick to respond to a few fashionable dinners or to a period of careful dieting, as the case might be. A period of indulgence in indiscriminate food brought back the sensitiveness and made her conscious of her teeth; on the other hand, a return to her rigid diet rendered them less sensitive, and enabled her to forget them.

Another interesting case of long standing adds testimony to the idea I have advanced. Over fifteen years ago I took a gentleman to Dr. Bronson to consult with him in reference to the advisability of filling certain eroding surfaces on his front teeth. Dr. Bronson, with his usual caution, advised me to wait and watch.

About this time the patient moved to the country, and after this the erosion seemed to be checked. As the years passed by I watched these teeth with great interest, and, as the erosion did not progress, I was taught the important lesson that it is not always well to cut out and fill these places. I did not then understand the cause of the erosion.

Recently I have learned from the gentleman that as long ago as the period I mentioned he had marked symptoms of gout, which he inherited, and that he had been put under treatment to counteract the uric-acid tendency, and that it was kept down only by most rigid care in diet, which he continues to this day. He also had eczematous troubles, which were cured by this anti-gout treatment.

If this case does not add testimony in support of my theory, it is worthy of relation, as it proves that erosion does not always progress, whatever the cause of its progression or of its arrest may be.

But I will not take your time by the relation of more cases.

Now, to recapitulate:

I have stated that I believe uric acid in the blood is the cause of these conditions, but, of course, I have no proof of its presence in the mouth, or of its being absolutely the very acid that causes the trouble; but I feel certain that there is ample proof that that derangement of the liver and kidneys, of which uric acid in the blood is the evidence, is the underlying condition that gives rise to the acid in the mouth, whatever that acid may be. It may not matter, after all, what the acid is, if we are correct in concluding that its origin is to be found in the faulty action of the liver and the kidneys. Once let this point be firmly established, and we will be able to indicate the means of cure.

As we all know, it is an easy matter with the litmus paper to determine the presence of an acid. It is quite a different and more difficult thing to tell what the acid is.

Aside from the observations of cases, it seems to me one of the strongest proofs of the truth of this general proposition is that I have many times been able to arrest this destructive action by correcting the diet and reforming the habits of the patients. This is not easy to do, as it is not often that one will have a patient who may be willing to conform to all the essential conditions.

I have already indicated what these conditions are. I am certain that in my own case much good came from the use of the lithia waters. Whether the benefit was derived from the minute proportions of lithia contained in them, or to the washing and dissolving action of the water itself, I am not able to say; but I incline to the latter belief, because I have also for years used the Poland water, which is said to be free from lithia, with as good result, as far as I could see, as that derived from the lithia water.

I have also used the Carlsbad and Bedford waters. These two waters give nearly the same analysis, and their action is supposed to be more particularly upon the liver.

If Sir Henry Thompson lays down the general proposition that after adult life is reached we eat too much, I think it may be also safely said we drink too little water. Aside from its washing action on the great organs of the body,—the liver and kidneys,—its influence is felt by the skin, which, in our dry climate and in our furnace-heated houses, becomes dry and hard and incapable of fulfilling its function of helping to eliminate the waste products through the medium of the insensible perspiration.

Another important factor in the prevention of this excess of uric acid is the oxygenation of the food-elements. This, of course, is accomplished by exercise in the open air, or by the adoption of the habit of deep breathing, as advocated in the Checkley System of Physical Culture. This latter is based upon the idea that the lungs are the boilers of the system, and that there cannot be complete physical development and perfect health without the full use of them.

Mr. Checkley says, "This truth lies at the very bottom of natural physical training. To learn to breathe is to learn the A, B, C of physical health. . . . The simplest preparatory exercise is full, long breathing.

"While standing or sitting in any proper attitude with the chest free, take in a long breath until the lungs seem full, taking care at the same time not to harshly strain the lungs or muscles. Hold the breath thus taken for a few seconds, and then allow it to slowly leave the lungs. By consciously breathing in this manner the lungs will be enlarged and strengthened, and the breathing will become slow. . . . The feeling of buoyancy given by this habit is not an illusion by any means. It is genuine."

In a very direct and natural manner, Mr. Checkley has here hit upon a great fundamental truth. The proper oxygenation of the food-elements must lie at the very foundation of good health.

Active exercise necessarily compels deep breathing. The laboring-man, working in the open air, breathes more or less deeply without knowing it, and retains his health without a thought or care. His food is completely burned up and converted into its equivalent of muscular force.

The rich man, in indolent ease, stuffs himself with tasty food, breathes only with a part of his lungs, gets drowsy, and goes to sleep, only to waken with a liver headache and creaking, snapping joints, and aching body, and with the conviction that life is not worth living. There can be no possible question of the advantage of deep breathing. Some day, like a pill or powder, it will be prescribed by physicians. It is so now, unconsciously, under the head of horseback riding, rowing, boxing, etc.

It need not be taken in this elaborate way. It does not require the paraphernalia of the athletic club-house or the home gymnasium. The habit can be formed, and no man can be so busy, or so rich, or so poor that he cannot, if he only will, frequently, and without the loss of a moment of time, expand his chest and take into his lungs large quantities of oxygen, on which his very life depends. If he has been overfed, it will be his only hope. If he has been underfed, it will help to keep him alive.

It may seem that I am wandering from my subject. If my theory of the cause of erosion is true, it must be seen that I am only indicating a most important factor in its cure.

There remains one other to be mentioned, and that is rest.

If a neurotic condition is sometimes primarily one of the factors in producing the uric-acid tendency, then the importance of rest is clearly seen. An overworked, nervous man, whose occupation keeps him in touch with the innumerable activities of this electrical age, and who develops the uric-acid tendency, followed by erosion of the teeth, may need to be told to rest.

An overfed, indolent woman, with an engorged liver and a consequent uric-acid tendency, must be told to exercise.

In conclusion, let me say, if this theory does not account for all of the facts observed in erosion, I shall join Dr. Darby in submitting it to the profession, with the confident belief that it will account for more of them than any that has yet been put forward.

